# Antidepressant and Anxiolytic Effects of Fermented Huauzontle, a Prehispanic Mexican Pseudocereal

**DOI:** 10.3390/foods12010053

**Published:** 2022-12-22

**Authors:** Lourdes Santiago-López, Arantxa Almada-Corral, Hugo S. García, Verónica Mata-Haro, Aarón F. González-Córdova, Belinda Vallejo-Cordoba, Adrián Hernández-Mendoza

**Affiliations:** 1 Laboratorio de Química y Biotecnología de Productos Lácteos, Centro de Investigación en Alimentación y Desarrollo A.C. (CIAD, A.C.), Carretera Gustavo Enrique Astiazarán Rosas, No. 46, Hermosillo 83304, Sonora, Mexico; 2 Unidad de Investigación y Desarrollo de Alimentos, Instituto Tecnológico de Veracruz, M.A. de Quevedo 2779, Col. Formando Hogar, Veracruz 91897, Veracruz, Mexico; 3 Laboratorio de Microbiología e Inmunología, Centro de Investigación en Alimentación y Desarrollo A.C. (CIAD, A.C.), Carretera Gustavo Enrique Astiazarán Rosas, No. 46, Col. La Victoria, Hermosillo 83304, Sonora, Mexico

**Keywords:** huauzontle, depression, anxiety, fermentation, antioxidant activity, lipoxygenase activity

## Abstract

This study aimed to assess the potential antidepressant- and anxiolytic-like effects of huauzontle fermented by *Lactiplantibacillus plantarum* Lp22. The possible association between oxidative stress/inflammation biomarkers and unconditional behavioural tests was also evaluated. Red light-induced stress mice C57Bl/6 (n = 5 per group) received orally either fermented or unfermented huauzontle, diazepam or fluoxetine. A non-stressed group which received saline solution was also included. Then, anxiety-related and depression-related behaviour tests were performed; after that, blood and tissues samples were collected to determine oxidative stress/inflammation biomarkers. The mice receiving both fermented and unfermented huauzontle spent more time (94 s) in open arms in the elevated plus maze test *p* < 0.05; besides, travelled longer distance (*p* < 0.05) and increased by more than 50% the exploration time for the open field, as well as the time spent in the illuminated zone (197 s) in the light/dark test. Furthermore, reduced immobility time in the tail suspension and forced swim tests (23.1 and 15.85, respectively), and anhedonia was no detected in the sucrose preference test. The oxidative stress index was lower in the liver of fermented huauzontle-treated mice, while enhanced levels of IL-10, MCP-1 and BDNF in plasma, and lipoxygenase (LOX) activity in the hippocampus were found. Finally, PCA revealed a positive correlation among LOX and BDNF and parameters determined in the anxiety tests, as between catalase activity and immobility time in the depression test. These findings indicate the novel potential therapeutic applications of fermented huauzontle on depression and anxiety-like behaviours possibly mediated by antioxidant and anti-inflammatory mechanisms.

## 1. Introduction

Huauzontle (*Chenopodium berlandieri* spp. *Nuttalliae)* is a pseudocereal native from Mexico, which has been a staple food since pre-Hispanic times. Like amaranth and quinoa, huauzontle is rich in dietary fiber, proteins, essential amino acids, fatty acids, mineral, phenolic compounds and vitamins [1]. Despite of this, it has been little explored from the perspective of functional foods. One of the first attempts made in this direction was carried out by Sánchez-Villa et al. [2], who supplemented tortillas with proteins isolated from the scarlet runner bean and huauzontle flour. Supplementation increased the nutritional value of the tortilla without significantly affecting their rheological and sensory features. In a related work, Garcia et al. [3] developed a fermented huauzontle-based beverage by using epiphytic bacteria from the same pseudocereal. This beverage showed in vitro antioxidant and anti-inflammatory properties, mainly attributed to phenolics and bacteria content. These properties, along with intrinsic health benefits of macro and micronutrients in plant foods may promote a protective effect against diseases related to oxidative stress and inflammation [4]. This becomes relevant considering that chronic inflammation and oxidative stress have been implicated not only in the pathophysiology of a number of chronic medical conditions (e.g., cancer, cardiovascular diseases, joint disorders, atherosclerosis, and ageing), but also in major neurological (e.g., Alzheimer’s, Parkinson’s) and mental disorders such as depression and anxiety [5,6]. Particularly, during the COVID-19 pandemic, an increase in depressive (26.7%) and anxiety (25.6%) disorders was recorded [7]; although, previous to the pandemic, mental health disorders were already a worldwide public health problem [8]. It has been documented that during brain-related disorders excessive production of reactive oxygen and nitrogen species (ROS and RNS, respectively) and pro-inflammatory cytokines (IL-6, TNFα, IL-1β), as well as down-regulation of Brain-derivate Neurotrophic Factor (BDNF) may occur caused by several reasons, including but not limited to: susceptibility of neuronal membranes fatty acids to lipid oxidation, autoxidation of several neurotransmitters and to the inappropriate microglia activation [9]. Hence, oxidative stress and inflammation have been considered crucial components of the pathogenesis of brain-related disorders, including anxiety-like and depression-like behaviours [10]. Despite the combined use of pharmacological and non-pharmacological interventions have been recommended by international guidelines, patients may prefer non-pharmacological options (e.g., herbal remedies) caused by either lack of efficacy or intolerable side effects of some medical treatments [11]. Non-pharmacological treatment approaches include, among others, psychological interventions (e.g., cognitive behavioural therapy or person-centered therapy), and integrated plans using healthy lifestyles, exercise, sleep and the influence of diet. In this last regard, particular interest has been centered on popularly acclaimed herbs and plant-based food materials with brain-health–promoting effects, especially those with high phenolic content and other bioactive compounds [12,13]. The large body of evidence in favour of plant-centered dietary patterns, has resulted in attempts to modify available plant-based foods to offer higher health benefits. Thus, considering that fermentation may boost the well-known benefits of a wide variety of food plant materials by influencing the bio accessibility of bioactive compounds immersed within the food matrix and their functional properties [14]; hence, in view of huauzontle has proteins, iron, calcium, phosphorus and vitamins A, B complex, C, which contribute to the proper functioning of the brain [15], this study aimed to assess the potential antidepressant- and anxiolytic-like effect of huauzontle fermented by *Lactiplantibacillus plantarum* Lp22 in an in vivo animal model. The possible association between oxidative stress/inflammation biomarkers and unconditional behavioural tests was also evaluated.

## 2. Materials and Methods

### 2.1. Bacterial Culture and Preparation of Fermented Huauzontle

*Lactiplantibacillus plantarum* Lp22, was previously isolated from huauzontle inflorescences, identified by DNA-based techniques, assessed for probiotic properties (unpublished data) and used to prepare a functional fermented beverage [3]. The bacterium was grown in MRS broth (de Man, Rogosa, and Sharpe, BD Difco, St. Louis, MO, USA) for 24 h and sub-cultured twice at 37 °C, 18 and 12 h. Bacteria from the last sub-culture were recovered by centrifugation (4500× *g*, 10 min, 10 °C), washed with phosphate buffer saline (PBS; 0.1 M, pH 7.2) and resuspended to an OD_600 nm_ = 0.5 (10^8^ CFU/mL).

Huauzontle florets were obtained from a local supermarket in Hermosillo, Sonora, (México) and processed as described by Garcia et al. [3] with slight modifications. Briefly, huauzontle inflorescences (0.3%, *w*/*v*) were washed with tap water, dried in a convection oven, and then steeped at 85 °C during 20 min. After cooled, fermentation was conducted without removing the inflorescences: 1% of the bacteria was inoculated and incubated at 37 °C during 24 h. Fermentation was stopped by quick chilling; subsequently, organic solids were removed by centrifugation (4500× *g*, 10 min, 10 °C) and the fermented liquid was stored in the dark at −20 °C until used.

### 2.2. Experimental Animals

Thirty male C57Bl/6 mice (weight 25–30 g, six weeks old), obtained from BIOINVERT (Mexico City, Mexico), were housed under controlled conditions of light (12/12 h cycles), temperature (23 ± 2 °C) and humidity (55 ± 5%). Animals were adapted to the environment 7 days before the study started (adaptation period). Animals were provided with a standard diet (Abene 7100 maintenance, Bioinvert, Mexico City, Mexico) and purified water *ad libitum*. All experiments using these mice were reviewed and approved by the Bioethics Committee of CIAD, A.C. (Protocol approval number CEI-017-1/2021) and were performed in strict accordance with the recommendations of the National Research Council guide for the Care and Use of Laboratory Animals [16].

Efforts were made to reduce the number of animals used; thus, the total number of animals was determined considering the equation reported by Arifin and Zahiruddin [17]. Clinical studies have evidenced that consumption of 250 mL of herbal infusions may have antidepressant and anxiolytic effects [18]. Hence, this volume was used to adjust the final amount of fermented or unfermented huauzontle infusion that was administered to mice weighing near 30 g.

### 2.3. Induced Stress Mice and Behavioural Testing

**Figure 1:**
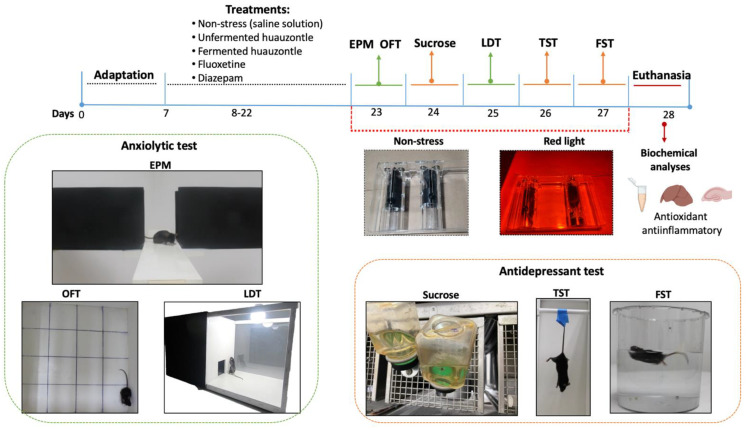
Schematic representations of the experimental protocol indicating the treatments and anxiety-like and depression-like tests. (Day 23) Elevated Plus Maze (EPM), Open Field Test (OFT), and sucrose preference test, (day 25) Dark/light exploratory box (LDT), (day 26) Tail suspension test (TST), (day 27) Forced swim test (FST). Day 28, mice were euthanized, via intraperitoneal injection with sodium pentobarbital (90 mg/kg body weight) and samples of blood, liver and hippocampus were collected for further analyses.

After a 7-day adaptation period, the animals were distributed into six equal groups (n = 5) using a simple randomization procedure (computerized random numbers). Basal glutathione reductase (GPx) activity and IL-6 cytokine level determined in blood samples were used as randomization variables. The treatment groups were distributed as follows: G1 = non-stress (sterile saline solution 1.5 mL p.o), G2 = chronic stress (sterile saline solution 1.5 mL p.o); G3 = anxiolytic positive control (Diazepam, 5 mg/kg p.o); G4 = antidepressant positive control (Fluoxetine, 10 mg/kg p.o); G5 = unfermented huauzontle (1.5 mL p.o); G6 = fermented huauzontle (1.5 mL p.o). The saline solution, fermented and unfermented huauzontle infusions were applied daily for 15 days, while diazepam and fluoxetine were administered 30 min before stress induction. The schematic procedure is shown in Figure 1. Diazepam (fast-acting benzodiazepine) and fluoxetine (selective serotonin reuptake inhibitor) are often considered drugs of choice due to their neuroprotective and anti-inflammatory effects on different experimental models. The concentrations used were determined based on previous reports [19,20].

Chronic stress was induced in all groups, except in G1, by exposing experimental animals individually to red light (LED lamp, 13.5 W 50/60 Hz, 60° angle, Tecnolite, Zapopan, Jalisco, Mexico) during 60 min prior to each behavioural testing began [21,22]. For that, each mouse was placed into a transparent acrylic mouse retainer (10 cm × 2.5 cm). The tail of the mouse was put into the lower opening of the back cover; next, the front sliding plug was adjusted to provide a comfortable space to the mouse, and then was kindly tighten. The mouse was then exposed to the red light located 25 cm over the animals. After the exposure time, mice were immediately subjected to different behavioural tests in a staggered manner i.e., from least stressful to the most stressful assay (Figure 1). Control animals (G1, non-stressed) were exposed to standard cool white LED light (5.5 W, 430 Lumens, 60 Hz, Tecnolite).

Induced stress and behavioural testing were conducted in separate rooms, respecting a schedule of 10:00 a.m. to 17:00 p.m. Images of the tests were analysed using the ToxTrac v2.61 software [23]. During all experiments, lighting was controlled, and white noise was used to mask background noises. All tests were recorded with a camera EOS 200D-800D Digital Rebel (Canon, Inc. Tokyo, Japan) and GoPro Hero7 Black camera (GoPro, Inc., San Mateo, CA, USA) located at a distance of 30 to 60 cm test-dependent. 

#### 2.3.1. Anxiolytic Evaluation

Elevated Plus Maze (EPM). EPM test is based on the rodents’ innate proclivity toward dark and enclosed spaces (approach), and unconditioned evasion to open and elevated spaces (avoidance) [24]. For this assay, the device was made with two opposing open arms (30 × 8 cm) and two opposing closed arms (30 × 8 × 15 cm), both connected by a central platform (8 × 8 cm) and elevated 120 cm above the floor. On day 23, the mice were placed at the midpoint of the apparatus. The time spent in the open arms were recorded during 5 min. A decrease in waiting time in open arms is considered as an anxiolytic behaviour [25]. 

Open Field Test (OFT). On day 23, the mice were placed in the central part of a wooden box (50 w × 50 l × 50 h cm) divided into 16 squares. During six minutes, the first minute for adaption, the behaviour was recorded [26]. Exploration rate (%), total distance travelled, faecal boli counts, and number of crossings into inner zone were monitored.

Light/dark transition test (LDT). LDT is based on the mice aversion to illuminated areas and spontaneous exploratory behaviour in response to a novel illuminated environment [27]. For this test, a box with two compartments, a dark zone and an illuminated zone (LED light 800 Lumens, Tecnolite) with dimensions of 30 × 28.5 × 33 cm, respectively, was used. The two compartments were connected by a door (7.5 × 7.5 cm). The mice were allocated into dark and the time between zones was recorded for 10 min. The time spent in the bright zone were recorded [28].

#### 2.3.2. Antidepressant Evaluation

Tail Suspension Test (TST). TST measure the desperation when mice are subjected to an uncomfortable situation from which they cannot escape. The mice were suspended 50 cm above the floor by adhesive tape placed 1 cm from the tip of their tail. The mice behaviour was recorded for 5 min after one minute of habituation period. The mice were considered immobile only when they hung passively and completely motionless [25]. 

Forced Swim Test (FST). For this test, the mice were individually allocated into a tall water-filled transparent cylinder (23 of height and 22 cm in diameter), with enough water (23 °C) so that mouse could not touch the bottom with its hind paws or its tail. The mice were forced to swim a 6-min session, divided into pre-test (the first minute) and test (the last 5 min). Water was changed after every session to avoid any influence on the next mouse. The test was recorded, and the mice were considered immobile when they remained floating motionless in the water. A decrease in the duration of immobility was indicative of an antidepressant-like effect [29]. 

Sucrose preference test. This test is characterized as the inability to experience pleasure from rewarding or enjoyable activities and is a core symptom of depression in humans [30]. For this assay, mice were given access to a 1% (*w*/*v*) fresh sucrose solution. Each animal had two bottles: one with the sucrose solution and the second with purified water. The position of the water bottles was changed 12 h later to avoid side preference effects. The consumption (mL) of each solution was measured 24 h after the bottles were placed. Sucrose preference was calculated as: [sucrose consumption/(sucrose consumption + water consumption) ∗ 100]. The percentage reduction in interest in a reward was considered a depressive behaviour [30].

### 2.4. Sample Collection

All the animals were euthanized at the end of the behavioural experiments. Blood samples were collected by cardiac puncture and placed in tubes with ethylenediaminetetraacetic acid (Vacutainer, K_2_EDTA, BD, Franklin Lakes, NJ, USA). Subsequently, plasma was obtained by centrifugation of the sample (1500× *g*, 10 min, 4 °C). Additionally, the liver and hippocampus were collected. The hippocampus was homogenized (1:5) in Tris-HCl (50 mM, pH 7.4), centrifugated (2500× *g*, 10 min, 10 °C) and the supernatant were stored at −80 °C until used [21,25]. The liver was washed and homogenized (10% *w*/*v*) (Bio-Gen Pro2000 Homogenizer, ProScientific, Oxford, CT, USA), in Tris-HCl (50 mM, pH 7.4) at 4 °C during five min; then, supernatants were collected (3000× *g*, 15 min, 10 °C) [31]. All samples were stored at −80 °C until used. The protein content in samples was determined by the DC protein Assay kit (Bio-Rad Laboratories, Hercules, CA, USA). The supernatant samples from liver and hippocampus were used to determine antioxidant capacity (ABTS and ORAC), catalase activity (CAT), malondialdehyde (MDA), glutathione peroxidase (GPx), hydrogen peroxide production, and Oxidative Stress index (OSi). Cytokines and brain derived Neurotrophic Factor (BDNF) were determined in samples of hippocampus and plasma, while lipoxygenase (LOX) activity was determined only in samples of hippocampus. 

#### Determination of Biomarkers Associated with Oxidative Stress and Inflammatory Response

Antioxidant capacity. Samples of the liver and hippocampus were evaluated by ABTS and ORAC methods following the procedure reported by Cuevas-González et al. [32]. Trolox (6-hydroxy-2, 3,7,8-tetra-methylchroman-2-carboxylic acid) was used as reference and calibration curve was prepared using a range from 0 to 500 μM). The results were expressed as μM of Trolox Equivalents. 

Enzymatic antioxidant activity. GPx activity was determined by measuring the oxidation of NADPH in presence of exogenous glutathione, 3,5, di-tert-4-butylhydro-xytoluene, and glutathione reductase [33]. The results were expressed as μM∗min∗mg of protein. CAT activity was determined by monitoring the decomposition of H_2_O_2_ at 240 nm at 37 °C during five min. The results were expressed as U∗mg of protein∗min [34].

Lipid peroxidation was estimated in terms of malondialdehyde (MDA) production, following the methodology reported by Sinha et al. [35]. The MDA concentration (μM) was calculated using the molar extinction coefficient of 0.156 M^−1^cm^−1^. Hydrogen peroxide (H_2_O_2_) content was determined after reaction with potassium iodide (KI) as reported by Caetano et al. [36]. A KI standard curve of 5 points (0–14 nM) was used to calculate the amount of H_2_O_2._

Oxidative Stress index (OSi). This parameter was calculated by combining data of the total oxidant status, represented by H_2_O_2_ levels, and the total antioxidant activity, represented by the value obtained in ABTS for each sample, as reported by Motor et al. [37] using the following equation:

OSi = Total oxidizing status (μM H_2_O_2_ Eq/L)/Total antioxidant capacity (μM Trolox Eq/L)

*Cytokines quantification*. Cytokines (IFNγ, IL-6, IL-10, IL-12, MCP1, TNFα) were determined in the hippocampus and plasma by flow cytometry (BD FACSCantoTM II, San Jose, CA, USA) using the mouse inflammation kit Cytometric Bead Array kit (CBA kits BD). The dates were analysed used the FlowJo software^TM^ (v10.8.1, BD). 

*Lipoxygenase inhibition assay*. The LOX activity was determined following the method reported by Garcia et al. [3], with some modifications. Briefly, the reaction mixture was comprised by the sample (10 μL), borate buffer (970 μL), and linoleic acid (10 μL) as reaction starter. Lipoxidase (10 μL, final concentration 20,000 U/mL) from *Glycine max* (Soybean) type-1B, (Sigma-Aldrich, St. Louis, MO, USA) was used as control. The inhibition of formation of conjugated dienes at 234 nm, after incubation for 5 min at 37 °C, was calculated using the following equation:

Inhibition (%) = [(absorbance of control − absorbance of sample)/absorbance of control] × 100.

BDNF measurement. The quantitative detection of BDNF in plasma and tissue homogenate was performed by using the mouse BDNF ELISA kit (Wuhan Fine Biotech Co., Ltd., Wuhan, China). Total protein of plasma and hippocampus samples were adjusted to <0.3 mg/mL and then analysed following the procedure set by the manufacturer.

### 2.5. Statistical Analysis

All values are expressed as mean ± standard deviation or error. All data were analysed with normality test using the Shapiro-Wilk test. Normal data were analysed using one way-ANOVA and Fisher test for multiple comparisons; while non-normal data were analysed using the non-parametric Kruskal-Wallis, and Dunn’s test for multiple comparisons, considering *p* < 0.05. All statistical tests were conducted using the GraphPad Prism software (v8.2.1, San Diego CA, USA). Principal Component Analysis (PCA) was performed in the Minitab software version 18.1.1 (Minitab, LLC, State Collage, PA, USA) in order to find any underlying correlations that could exist in the set of variables.

## 3. Results and Discussion

### 3.1. Locomotor Activity and Reduced the Anxiety-like Behaviour

**Figure 2:**
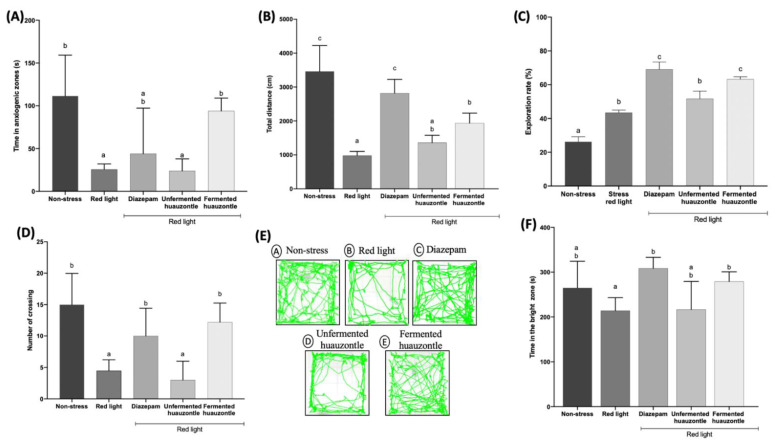
Effect of huauzontle on anxiety-related behaviour in the Elevated Plus Maze (EPM), Open Field Test (OFT) and Dark/light Transition Test (LDT). (**A**) Time in anxiogenic zones for EPM test; (**B**) Total distance travelled; (**C**) exploration rate; (**D**) number of crossings; (**E**) Representative imagen of OFT test. (**F**) Time in the bright zone for dark/light zone. Different letters indicate statistical difference among groups, EPM data analysed by Kruskal-Wallis and the date are expressed as median and IC90 for EPM. OFT (**B**–**D**) and dark/light analysed by parametric statistics (one-way ANOVA) with *p* < 0.05. Data are expressed as mean ± SEM (n = 5).

In the EPM test, data showed that animals exposed to red light (stressed) spent less time (*p* < 0.05) in the anxiogenic zone, indicative of fear, associated to symptoms of anxiety [38]. However, the stressed animals administered with fermented huauzontle showed an increased activity in open arms, similar (*p* < 0.05) to those of animals of the control group (non-stressed) and the anxiolytic positive control group (Diazepam), which reflects an anti-anxiety behavior (Figure 2A). Conversely, no anti-anxiety effect was observed in stressed animals administered with unfermented huauzontle.

Furthermore, the application of huauzontle unfermented and fermented for 15 days modified the locomotor activity (Open Field Test) of mice. The stressed animals administered with fermented huauzontle showed greater distance travelled, similar to the non-stressed animals, and different (*p* < 0.05) to those stressed (Figure 2B). Additionally, a number of crossings, faeces, and exploration rate were analysed. The results suggest that stressed animals fed with unfermented and fermented huauzontle had a higher (*p* < 0.05) exploration rate (51.69% and 63.25%, respectively), compared to the stressed animals (43.45%) (Figure 2C). Furthermore, it was observed that the stressed animals administered with fermented huauzontle performed a greater number of crossings to the inner zone (Figure 2D), which can be pictured in the images of Figure 2E. Overall, in all treated groups, except for the non-stressed group, there was an average of 1 to 3 faecal boli during the test time and showed no statistical difference (*p* > 0.05) among groups (Figure S1). The lower the number of crossings through the central zone of the box, and the higher the thigmotaxis state of the mice is associated with an increase in anxiety levels [26]. In the representative images of each study group (Figure 2E), it can be observed that the mice administered with huauzontle showed a greater number of crossings towards the central area, with similar results to the group treated with diazepam. Additionally, considering that in an episode of anxiety, events of defecation increased, we observed on average 2 faecal boluses from the non-stressed group and those groups treated with huauzontle and diazepam, and 4 boluses for the stressed group, but no differences were found (*p* > 0.05). 

For the LDT test, the anxiolytic effect was measured by the time spent in the illuminated zone [27]. In this sense, there were no observed differences (Figure 2F) among non-stressed and red light-stressed animals (*p* > 0.05); however, mice treated with either the anxiolytic or huauzontle, particularly with the fermented plant, showed higher (*p* < 0.05) spend time at the illuminated zone (>197 s) comparted to red light stressed animals. Anxiety produces freezing, elevated heart rate and decrease food consumption. Besides, mice with anxiety-like behaviour tend to prefer dark and enclosed spaces as a natural instinct to reduce risk of predation [39]. All these anxiety-like behaviours are regulated by bed nucleus of the stria terminalis the brain. This circuit is important in the stimulation of hypothalamic-pituitary-adrenal axis and is of interest in human stress-related psychiatric diseases like anxiety [40]. Overall, our findings suggest a potential anxiolytic effect of huauzontle; however, further studies are needed to determine the possible mechanisms underlying this effect. 

Dietary fibre, proteins and essential amino acids, phenolic compounds as well as minerals and vitamins are some of the components of huauzontle identified to exhibit health benefits [3]. Hence, considering the increasing evidence regarding the effect of trace elements on brain and behavioural function [41], it is reasonable to believe that a less stressful effect on animals administrated with huauzontle may be associated, at least in part, to such trace elements present in huauzontle.

### 3.2. Depressive-like Behaviour Mice

**Figure 3:**
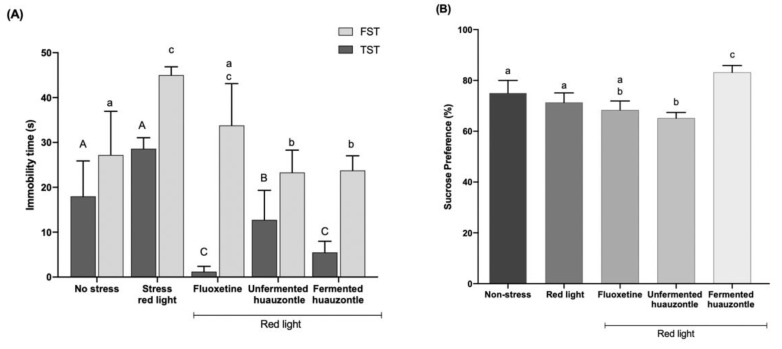
Effect of huauzontle on depressive-like behaviour. (**A**) Immobility time for FST and TST test; (**B**) Sucrose preference (%). Data are expressed as mean ± SEM (n = 5), *p* < 0.05. Capital letters indicate statistical difference for immobility time for TST test, and lowercase letters denote difference for the FST test and sucrose preference.

The immobility times recorded in both TST and FST are depicted in Figure 3A. The immobility time observed in the TST was not different (*p* > 0.05) between animals from the non-stressed and stressed groups, while the treatments with fermented and unfermented huauzontle resulted in a significant (*p* < 0.05) reduction (15.85 s and 23.1 s, respectively) of the immobility time. Particularly, the fermented huauzontle group was not different to the antidepressant positive control (Fluoxetine) group. The same effect was observed at FST test, were red light stressed animals showed the higher immobility times (*p* < 0.05), followed by the Fluoxetine treatment; even fermented and unfermented huauzontle treatments showed a shorter (*p* < 0.05) immobility time than the antidepressant positive control group, then, a higher immobility time indicates a greater depressive state [42,43]. Hence, our data suggest that huauzontle preparations interact with chronic stress to modulate their effects on mice, providing evidence for their potential antidepressant-like effect. Although the components of fermented huauzontle that might be involved are still unknown, some studies have documented that fermenting bacteria, including probiotics, can produce some neuroactive compounds (e.g., acetylcholine, histamine, serotonin, dopamine and gamma-aminobutyric acid), which may help to prevent some psychological disorders [44], probably, through modulation of microbiota, the central nervous system, and the immune system. Furthermore, studies have revealed the beneficial effects of phytochemicals (e.g., polyphenols, ferulic acid, proanthocyanidin, and quercetin) on depression [3,45]. In this sense, it has been recognized that huauzontle is an important source of polyphenols, although these have not been characterized yet. Moreover, a previous study has suggested that the huauzontle-fermentation process could improve the release of bound phenolic acids [3], which may contribute to the effects found in the behavioural tests.

On the other hand, no differences were found (*p* > 0.5) in the preference for sucrose among the groups evaluated, except for those administered with fermented huauzontle, which exhibited the highest (*p* < 0.5) percentage of preference (83.51%) (Figure 3B). It has been demonstrated that several stressors substantially reduce the preference for sucrose solution, but treatment with antidepressants restores the preference for sucrose [30]. Thus, our results imply that the administration of huauzontle decreases anhedonia. Therefore, these data help to complement the findings described above. 

### 3.3. Biochemical Parameters 

The antioxidant activity in liver samples was not different (*p* > 0.05) by the ORAC method among treated groups, but a significant higher activity was observed, in liver samples of group administered with fermented and unfermented huauzontle by ABTS. In contrast, the antioxidant activity in hippocampus samples was not different (*p* > 0.05) by ABTS among treated groups; however, a significant (*p* < 0.05) higher activity was observed by ORAC method in hippocampus samples of group administered with huauzontle, particularly from fermented one. Regarding CAT and GPx activities, no differences (*p* > 0.05) were found in liver samples among treated groups. While in hippocampus samples, animals treated with huauzontle showed a reduced (*p* < 0.05) CAT and GPx activities, compared to red light-stressed and non-stressed groups. Overall, no statistical differences were found among groups in MDA level, except for the liver sample at the non-stressed group, which showed the lowest (*p* < 0.05) MDA value. It should be noted that animal receiving fermented huauzontle had a significant (*p* < 0.05) lower OSi value than red light stressed mice, and similar to non stressed mice (Table 1). Despite data showed no significant differences on antioxidant enzymes and antioxidative capacity, the OSi was equal to the non-stressed group, indicating the release of antioxidant compounds during fermentation, which regulate the stress generated in the mice. In this sense, besides the possible release of bound phenolic acids described above, it has been reported the intracellular content of probiotics, may be bioavailable to regulate antioxidant status by reducing hepatic mitochondrial dysfunction in rats, thus providing a protective effect by significant lower OSi [31]. 

Oxidative stress caused by ROS, reactive nitrogen species, and unbound, adventitious metal ions has been associated to several neurodegenerative diseases. Antioxidants may prevent and/or help in the treatment of depressive disorders, which has led to the exploration of antioxidant agents derived from plants [3]. Hence, considering that ROS production may be maintained in equilibrium by the presence of antioxidant compounds (e.g., phenolic compounds, and probiotic bacteria) in fermented huauzontle; therefore, its intake may decrease the OSi at the hippocampus level, thus, ROS generation in the brain can be kept in balance and consequently avoid oxidant-generated cell damage, protein damage, lipid peroxidation and unaltered neuronal function and brain activity [46].

Additionally, inflammation is a crucial component in anxiety and depression development. An increase in pro-inflammatory cytokines and oxidative stress in the brain and peripheral immune system contributes to the behavioural alterations associated to major depressive disorders [47]. Hence, reducing inflammation and oxidative stress may play a key role in neuroprotection. Our data showed a significant increment (*p* < 0.05) in anti-inflammation biomarkers (IL-10, and MCP1) in animals receiving fermented huauzontle, compared to red light-stressed animals and animals receiving unfermented huauzontle. In particular, IL-10 and MCP-1 were significantly higher in plasma in the group treated with fermented huauzontle; meanwhile, no difference among groups were observed in the hippocampus (Table 2). Data corresponding to IFNγ, and IL-6 INF are not shown, as these were found to fall below the detection limit of the kit employed (5 and 2.5 pg/mL, respectively). Anxiety and depression behaviours are accompanied by up-regulation of pro-inflammatory cytokines (TNFα, IL-6, IL-1β), which can be produced by neurons, astrocytes and microglia [48]. On the other hand, peripherally cytokines can enter the brain, and signalling pathways can be activated by the Toll-like receptor-4 (TLR4) and NOD-like receptor and activate the nuclear factor kappa-B (NF-kB). Some foods rich in phenols, flavonoids, and probiotics can inhibit TLR4 signalling [49]. This effect could be correlated to the consumption of fermented huauzontle rich in phenolic compounds, and the presence of potentially probiotic bacteria, although no significant differences were noted in the hippocampus. We observed a higher concentration of IL-10 (regulatory cytokine) in plasma of the group treated with fermented huauzontle and of MCP-1 in both groups, showing a significant difference with the stressed and non-stressed groups. Although there are no reported studies on the composition of huauzontle and its effect on depression and anxiety, some other studies with plant extracts have shown that its consumption promotes the up-regulation of pro-inflammatory cytokines, low expression of TLR4/NLRP3 inflammasome pathway in the brain of mice, thereby improving the depression/anxiety-like behaviour [50]. In particular, in our study, MCP-1 was significantly highest in the fermented huauzontle group, and recent findings have implicated chemokines (small molecules 8–12 kDa) in neuroimmune processes and have an important role in the modulation of neuroendocrine functions, cell adhesion, activation of macrophages, monocytes, dendritic cells and T-lymphocytes [51,52]. Some studies correlated the severity and pathophysiology of depression with the low concentration serum level of MCP-1 in patients diagnosed with some major depressive disorders [53,54,55,56]. Although the mechanisms of MCP-1 are still unclear in major depressive disorders, an enhancement of MCP-1 might be correlated with increased dopaminergic activity in the central nervous system [55].

**Figure 4:**
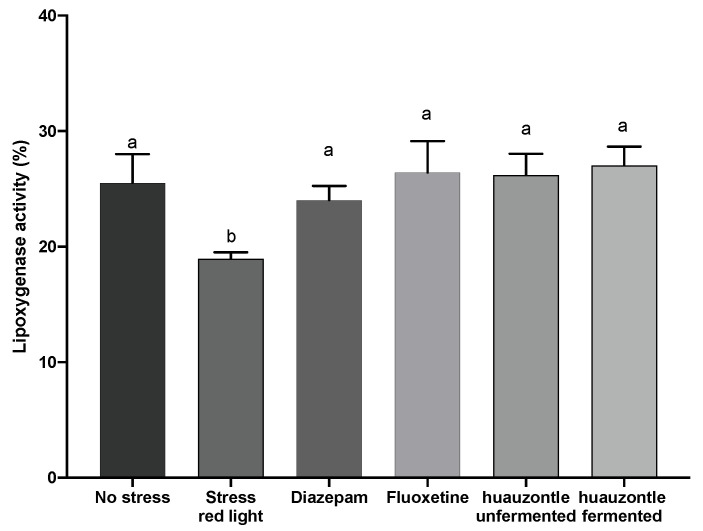
Lipoxygenase activity in samples of hippocampus. Different letters showed statistical difference among groups. Date shown the percentage ± DS, (n = 5), *p* < 0.05.

In addition, the inhibition of LOX activity increased for the groups administered with either fermented or unfermented huauzontle, and drugs, with values of 27.02%, 26.18%, 26.42%, and 24.03%, which contrasted with 18.95% of the stressed group (*p* < 0.05). The values of the treated groups were similar to the non-stressed group (Figure 4). Although LOX activity has been barely explored in depressive and anxiety disorders, this enzyme plays an important role in central nervous system functions [57]. The brain is particularly enriched with polyunsaturated fatty acids (PUFAs) represented by the omega-6 and omega-3 series. These PUFAs include arachidonic acid and docosahexaenoic acid, respectively [58]. Both fatty acids have a function in the synthesis of prostaglandins and leukotrienes (inflammatory molecules), the action of cyclooxygenases and lipoxygenases [59]. Previous studies have shown that inhibition or genetic disruption of 5-LOX has antidepressant effects in mice [60]. In particular, hippocampal 5-LOX enzyme is considered to play an important role in various pathophysiological processes in the central nervous system [61]. For instance, a study evaluated 5-LOX translocation in a model for depression in mice. The results showed that 5-LOX was positively located in astrocytes, and not positive in microglia. Thus, these results suggest that hippocampal astrocytes, are the primary cells responsible for 5-LOX activation.

Finally, the BDNF production was determined in plasma and hippocampus. Our results are displayed in Table 2. The data show that the administration of fermented huauzontle promoted the production of BDNF (*p* < 0.05); meanwhile, stressed and non-stressed mice had a lower level. Especially, for the hippocampus, only BDNF was detected in the same group and the non-stressed animals. The production of BDNF (neurotrophins), is vital to the survival, growth, and maintenance of neurons in the brain and then, has an important purpose in emotional and cognitive functions, probably driven by the downregulation of BDNF in the hippocampus contributes to the dysfunction of astrocytes in microglia [62]. Our results showed that stress was associated with reduced BDNF in the hippocampus, and the same results were observed in plasma. BDNF and neural cell adhesion molecules are the two best studied.

Recently, oxidative stress has been implicated in depression and anxiety, attributed to decreased plasma concentrations of antioxidants and antioxidant enzyme activities; also, increased levels of MDA and arachidonic acid [63]. A link between oxidative stress and emotional stress at brain level has been recognized, and further damages to the nervous system [64]. The brain is especially prone to oxidative stress because it has a high amount of transition metals and polyunsaturated fatty acids that provide a substrate for lipid peroxidation, in addition to its high oxygen consumption rate and limited antioxidant defences [63].

**Figure 5:**
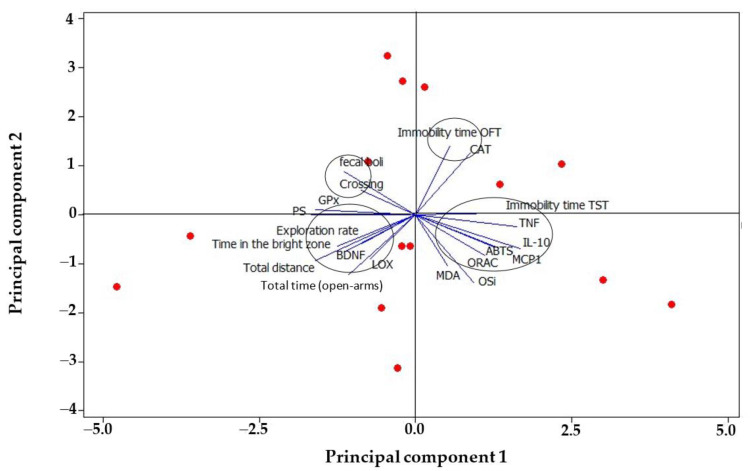
Analysis of principal components grouping biochemical variables and behavioural variables of depression and anxiety. CAT: catalase, OFT: Open Field Test, TST: Tail Suspension Test, BDNF: Brain-derived Neurotrophic Factor, OSi: Oxidative stress index, LOX: Lipoxygenase, PS: Preference Sucrose, GPx: glutathione reductase.

Finally, the PCA shows the grouping of the variables analysed (Figure 5). We can distinguish two groupings with most of the variables. For example, the variables MDA, OSi, IL-10, ORAC, MCP1 are positively clustered in PC1 and independent to the behavioural variables; meanwhile observe in PC2, the behavioural variables are negatively clustered with the anxiety-related variables and correlated with LOX activity and BDNF. This correlation suggests that huauzontle consumption may contribute to the suppression of prostaglandins (PG) production by inhibiting the enzymes cyclooxygenase and LOX, which are related to the synthesis of PG from free arachidonic acid (AA) in the hippocampus and, also, decrease the concentration of BDNF, which then increases under conditions of neuronal disorders. This is relevant considering that PG suppression may confer the alleviation of some brain diseases, including depression and anxiety.

Overall, the significance of oxidative stress and inflammation in neural disorders produces a growing interest in the supplementation with antioxidants from food that may be therapeutic to decrease the production of ROS and reduce symptoms of depression and anxiety [65]. In this sense, various plant products and herbal infusions have demonstrated antioxidant activities in vitro and in vivo through the presence of vitamins, essential oils, flavonoids, and phenolic compounds, which have the capacity to act as free radical scavengers, and inhibit lipid peroxidation, maintain in equilibrium the mitochondrial balance in the brain, protecting the morphological aspects and improve the functions of neurons [66]. Phenolic compounds, in particular, can mediate neuroinflammation by targeting TLR4 and inhibit the TLR4/NLRP3 inflammasome pathway; whereas flavonoids can pass through the blood barrier and interact with cell membrane and promote changes in the lipid head. Additionally, phenolic compounds, flavonoids, and bacteria are known for antioxidant properties, they block caspase-3 activation and decrease ROS, promoting the antioxidant protein expression (superoxide dismutase, CAT, GPx), glutathione synthesis, with amyloidogenic effects [65]. All these effects may be caused by the interaction of bioactive compounds and bacterial components with α-aminobutyric acid type A receptor in the central nervous systems, modulates Acetyl coenzyme A activity [67]. There are reports of antioxidant, and anti-inflammatory properties of huauzontle, particularly by the presence of phenolic compounds, which are supplemented by the presence of other components from bacteria, which demonstrated these same properties, and both can promote the regulation of inflammation by cytokines regulation and LOX activity [3].

## 4. Conclusions

To the best of our knowledge, this is the first study that documents the potential benefits of huauzontle consumption against depression-like and anxiety-like disorders. The findings in this study related to unconditioned behavioural tests (particularly TST and OFT) should help to enhance the value of this pre-Hispanic plant and show that the anti-inflammatory pathway (regulation of cytokines, LOX activity) is mainly associated with positive health effects. Additionally, the correlation between LOX activity and BDNF levels leads to hypothesize that huauzontle contains components, released by fermentation, which may participate in the intestine-brain axis to reduce symptoms of depression and anxiety. However, further studies are still needed to determine the specific activation pathways, mitochondrial function and the main bioactive compounds associated with these effects.

## Figures and Tables

**Table 1:** Antioxidant status evaluated in liver and hippocampus samples at the different study groups.

Liver						
**Treatments**	**ABTS**	**ORAC**	**CAT**	**GPx**	**OSi**	**MDA**
Non-stresse^d^	1512. 75 ± 76.37 ^a^	5102.76 ± 1068.48 ^a^	7.32 ± 2.00 ^a^	35.92 ± 13.60 ^a^	0.02 ± 0.01 ^b^	0.39 ± 0.23 ^a^
Red light	1435.64 ±134.66 ^a^	5189.70 ± 1786.08 ^a^	10.74 ± 2.86 ^a^	19.50 ± 2.12 ^a^	0.29 ± 0.11 ^a^	0.63 ± 0.14 ^b^
Unfermented huauzontle	1772.44 ± 77.42 ^b^	4411.40 ± 1313.12 ^a^	11.76 ± 4.47 ^a^	16.74 ± 6.28 ^a^	0.22 ± 0.14 ^a^	0.68 ± 0.12 ^b^
Fermented huauzontle	1707.27 ± 80.11 ^b^	4840.90 ± 182.04 ^a^	9.12 ± 0.93 ^a^	13.33 ± 2.32 ^a^	0.05 ± 0.18 ^b^	0.52 ± 0.19 ^b^
Hippocampus						
Non-stresse^d^	235.75 ± 71.54 ^a^	195.12 ± 85.27 ^ac^	1.16 ± 0.52 ^a^	2.61 ± 0.38 ^b^	0.26 ± 0.05 ^a^	0.75 ± 0.07 ^a^
Red light	210.60 ± 72.15 ^a^	157.3 ± 72.40 ^a^	1.09 ± 0.30 ^a^	2.29 ± 0.45 ^b^	0.27 ± 0.09 ^a^	0.69 ± 0.06 ^a^
Unfermented huauzontle	259.05 ± 85.12 ^a^	404.8 ± 53.09 ^b^	1.00 ± 0.39 ^ab^	1.57 ± 0.37 ^ab^	0.21 ± 0.11 ^a^	0.62 ± 0.15 ^a^
Fermented huauzontle	259.12 ± 63.08 ^a^	297.7 ± 65.76 ^c^	0.66 ± 0.13 ^b^	1.91 ± 0.77 ^a^	0.22 ± 0.08 ^a^	0.73 ± 0.04 ^a^

Different letters in each column indicate statistical difference among groups. Data are expressed as mean ± DS, (n = 5), *p* < 0.05. The data were analysed with parametric statics. ABTS and ORAC (μmol of TE); CAT (U/mg protein/min); GPx (μM/min/mg of protein); OSi (arbitrary units) MDA (μM/mg protein).

**Table 2:** Cytokines concentration, BDNF production in hippocampus and plasma.

	Plasma			
	**Cytokines (pg/mL)**			
**Treatments**	**IL-10**	**MCP1**	**TNFα**	**BDNF (pg/mL)**
Non-stressed	49.05 ± 3.96 ^a^	16.42 ± 5.33 ^a^	9.10 ± 2.29 ^a^	171.87 ± 63.13 ^a^
Red light	13.87 ± 1.80 ^b^	18.76 ± 1.17 ^a^	6.64 ± 1.45 ^a^	179.62 ± 37.71 ^a^
Unfermented huauzontle	10.69 ± 2.78 ^b^	24.56 ± 3.78 ^b^	6.34 ± 1.44 ^a^	191.11 ± 23.14 ^a^
Fermented huauzontle	24.97 ± 7.30 ^c^	27.02 ± 5.34 ^b^	5.92 ± 0.64 ^a^	232.20 ± 23.59 ^b^
Hippocampus				
Non-stressed	10.16 ± 2.94 ^a^	16.33 ± 1.79 ^a^	<7.3	75.00 ± 35.31 *
Red light	11.44 ± 3.14 ^a^	16.96 ± 2.38 ^a^	10.47 ± 0.86 ^a^	<18.75
Huauzontle unfermented	13.35 ± 2.64 ^a^	17.18 ± 1.54 ^a^	15.87 ± 4.40 ^a^	<18.75
Huauzontle fermented	10.62 ± 1.66 ^a^	16.43 ± 1.23 ^a^	10.81 ± 0.84 ^a^	77.80 ± 5.24 *

Different letters by column indicate differences among groups evaluated. Data are expressed as mean ± DS, (n = 5), *p* < 0.05. The data were analysed using parametric statistics. * The data were analysed using t-student independent sample.

## Data Availability

The data presented in this study are available on request from the corresponding author.

## Supplementary Materials

The following supporting information can be downloaded at: https://www.mdpi.com/article/10.3390/foods12010053/s1, Figure S1: Effect of huauzontle on anxiety-related behaviour on the number of fecal boli excreted. A moderate reduction on the fecal boli excretion, not statistically significant, was observed in huauzontle-treated mice.
