# Supplementary material for: Antidepressant and Anxiolytic Effects of Fermented Huauzontle, a Prehispanic Mexican Pseudocereal
Source: Foods. 2022 Dec 22;12(1):53. doi: 10.3390/foods12010053 (PMC9818389; doi:10.3390/foods12010053)
Supplement: Supplementary file 1 [file foods-12-00053-s001.zip › Supplementary material.pdf]

Supplementary material

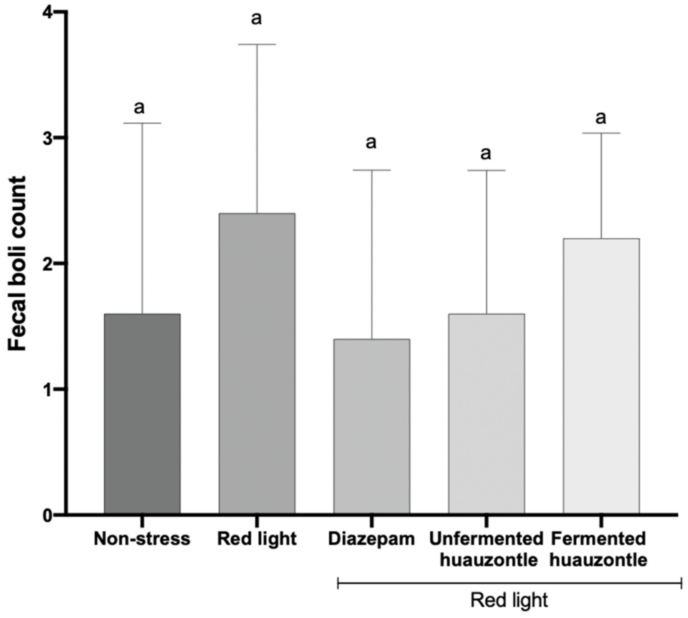

Figure S1. Effect of huauzontle on anxiety-related behaviour on the number of fecal boli excreted. A moderate reduction on the fecal boli excretion, not statistically significant, was observed in huauzontle-treated mice.
